# Supplementary material for: Crawling and Gliding: A Computational Model for Shape-Driven Cell Migration
Source: PLoS Comput Biol. 2015 Oct 21;11(10):e1004280. doi: 10.1371/journal.pcbi.1004280 (PMC4619082; doi:10.1371/journal.pcbi.1004280)
Supplement: S1 Code — (ZIP) [file pcbi.1004280.s012.zip › release/tst/doc/html/parse_8cpp.html]

Tissue Simulation Toolkit: parse.cpp File Reference


Tissue Simulation Toolkit
 0.1.4.1


- Main Page
- Namespaces
- Classes
- Files

- File List
- File Members

Functions

parse.cpp File Reference

`#include <stdio.h>`  
`#include <locale.h>`  
`#include <string.h>`  
`#include <stdlib.h>`  
`#include <errno.h>`  
`#include "warning.h"`  
`#include "parse.h"`  
`#include "output.h"`

Include dependency graph for parse.cpp:

|  |  |
| --- | --- |
| Functions | |
| char \* | ParsePar (FILE \*fp, char \*parameter, bool wrapflag) |
|  | |
| int | igetpar (FILE \*fp, char \*parameter, bool wrapflag) |
|  | |
| int | igetpar (FILE \*fp, char \*parameter, int default\_val, bool wrapflag) |
|  | |
| float | fgetpar (FILE \*fp, char \*parameter, bool wrapflag) |
|  | |
| float | fgetpar (FILE \*fp, char \*parameter, double default\_val, bool wrapflag) |
|  | |
| double \* | dgetparlist (FILE \*fp, char \*parameter, int n, bool wrapflag) |
|  | |
| char \* | sgetpar (FILE \*fp, char \*parameter, bool wrapflag) |
|  | |
| char \* | sgetpar (FILE \*fp, char \*parameter, const char \*default\_val, bool wrapflag) |
|  | |
| char \* | bool\_str (bool bool\_var) |
|  | |
| bool | bgetpar (FILE \*fp, char \*parameter, bool wrapflag) |
|  | |
| bool | bgetpar (FILE \*fp, char \*parameter, int default\_val, bool wrapflag) |
|  | |
| char \* | SearchToken (FILE \*fp, char \*token, bool wrapflag) |
|  | |
| int | TokenInLineP (char \*line, char \*token) |
|  | |
| void | SkipLine (FILE \*fp) |
|  | |
| void | SkipToken (FILE \*fp, char \*token, bool wrapflag) |
|  | |

## Function Documentation

|  |  |  |  |
| --- | --- | --- | --- |
| bool bgetpar | ( | FILE \* | *fp*, |
|  |  | char \* | *parameter*, |
|  |  | bool | *wrapflag* |
|  | ) |  |  |

References bgetpar().

Referenced by bgetpar(), and Parameter::Read().

|  |  |  |  |
| --- | --- | --- | --- |
| bool bgetpar | ( | FILE \* | *fp*, |
|  |  | char \* | *parameter*, |
|  |  | int | *default\_val*, |
|  |  | bool | *wrapflag* |
|  | ) |  |  |

References bool\_str(), error(), ParsePar(), and warning().

|  |  |  |  |  |  |
| --- | --- | --- | --- | --- | --- |
| char\* bool\_str | ( | bool | *bool\_var* | ) |  |

Referenced by bgetpar().

|  |  |  |  |
| --- | --- | --- | --- |
| double\* dgetparlist | ( | FILE \* | *fp*, |
|  |  | char \* | *parameter*, |
|  |  | int | *n*, |
|  |  | bool | *wrapflag* |
|  | ) |  |  |

References error(), ParsePar(), and warning().

Referenced by Parameter::Read().

|  |  |  |  |
| --- | --- | --- | --- |
| float fgetpar | ( | FILE \* | *fp*, |
|  |  | char \* | *parameter*, |
|  |  | bool | *wrapflag* |
|  | ) |  |  |

References fgetpar().

Referenced by fgetpar(), and Parameter::Read().

|  |  |  |  |
| --- | --- | --- | --- |
| float fgetpar | ( | FILE \* | *fp*, |
|  |  | char \* | *parameter*, |
|  |  | double | *default\_val*, |
|  |  | bool | *wrapflag* |
|  | ) |  |  |

References ParsePar(), and warning().

|  |  |  |  |
| --- | --- | --- | --- |
| int igetpar | ( | FILE \* | *fp*, |
|  |  | char \* | *parameter*, |
|  |  | bool | *wrapflag* |
|  | ) |  |  |

References igetpar().

Referenced by igetpar(), and Parameter::Read().

|  |  |  |  |
| --- | --- | --- | --- |
| int igetpar | ( | FILE \* | *fp*, |
|  |  | char \* | *parameter*, |
|  |  | int | *default\_val*, |
|  |  | bool | *wrapflag* |
|  | ) |  |  |

References ParsePar(), and warning().

|  |  |  |  |
| --- | --- | --- | --- |
| char\* ParsePar | ( | FILE \* | *fp*, |
|  |  | char \* | *parameter*, |
|  |  | bool | *wrapflag* |
|  | ) |  |  |

References error(), SearchToken(), and warning().

Referenced by bgetpar(), dgetparlist(), fgetpar(), igetpar(), and sgetpar().

|  |  |  |  |
| --- | --- | --- | --- |
| char\* SearchToken | ( | FILE \* | *fp*, |
|  |  | char \* | *token*, |
|  |  | bool | *wrapflag* |
|  | ) |  |  |

References errno, error(), ReadLine(), and warning().

Referenced by ParsePar(), and SkipToken().

|  |  |  |  |
| --- | --- | --- | --- |
| char\* sgetpar | ( | FILE \* | *fp*, |
|  |  | char \* | *parameter*, |
|  |  | bool | *wrapflag* |
|  | ) |  |  |

References sgetpar().

Referenced by Parameter::Read(), and sgetpar().

|  |  |  |  |
| --- | --- | --- | --- |
| char\* sgetpar | ( | FILE \* | *fp*, |
|  |  | char \* | *parameter*, |
|  |  | const char \* | *default\_val*, |
|  |  | bool | *wrapflag* |
|  | ) |  |  |

References ParsePar(), and warning().

|  |  |  |  |  |  |
| --- | --- | --- | --- | --- | --- |
| void SkipLine | ( | FILE \* | *fp* | ) |  |

References ReadLine().

|  |  |  |  |
| --- | --- | --- | --- |
| void SkipToken | ( | FILE \* | *fp*, |
|  |  | char \* | *token*, |
|  |  | bool | *wrapflag* |
|  | ) |  |  |

References error(), and SearchToken().

|  |  |  |  |
| --- | --- | --- | --- |
| int TokenInLineP | ( | char \* | *line*, |
|  |  | char \* | *token* |
|  | ) |  |  |


---

Generated on Thu Aug 14 2014 22:04:01 for Tissue Simulation Toolkit by  

 1.8.6
